# Australian and Pacific contributions to the genetic diversity of Norfolk Island feral chickens

**DOI:** 10.1186/1471-2156-14-91

**Published:** 2013-09-24

**Authors:** Shannan M Langford S, Spiridoula Kraitsek, Bruce Baskerville, Simon YW Ho, Jaime Gongora

**Affiliations:** 1 Veterinary Science, University of Sydney, Sydney, NSW 2006, Australia; 2 Kingston & Arthur’s Vale Historic Area, Norfolk Island Government, Kingston, NI 2899, Australia; 3 School of Biological Sciences, University of Sydney, Sydney, NSW 2006, Australia

**Keywords:** Norfolk Island, Mitochondrial DNA, Network analysis, Chickens, Ferals

## Abstract

**Background:**

Norfolk Island has a population of feral chickens which could be the result of domestic stock introduced onto the island by British settlers in 1788. However, there is ongoing debate about their origins because multiple human arrivals to the island may have brought chickens with them. Here we investigate the genetic origins of these feral chickens by sequencing their mitochondrial control region. We infer their phylogenetic relationships using a large dataset of novel sequences from Australian mainland domestic chickens and published sequences from around the world.

**Results:**

Eleven control region haplotypes were found among the Norfolk Island feral and Australian mainland domestic chickens. Six of the Norfolk Island haplotypes fall within haplogroup E, but given the worldwide distribution of this haplogroup, the putative European origin of these chickens requires further investigation. One haplotype common among Norfolk Island and Australian samples belonged to a subgroup of haplogroup D, which appears to be restricted to chickens from Indonesia, Vanuatu and Guam.

**Conclusions:**

Our data show that at least two mitochondrial DNA haplogroups (D and E) have contributed to the genetic make-up of Norfolk Island feral chickens. In addition, we have provided insights into the discrete geographical distribution and diversity of the chicken haplogroup D. In view of the worldwide interest in the characterisation of poultry resources, further assessment of chicken populations of Island Southeast Asia and the Pacific region is warranted.

## Background

Norfolk Island is an Australian territory situated between New Zealand and New Caledonia at 29°2’S and 167°56’E, with a land area of 3,455 hectares. In archaeological terms, it is considered to be one of the “mystery islands” of the South Pacific owing to its very isolated situation at the western extremity of Polynesian colonisation [1]. Polynesians settled and then abandoned the island during the 13th-15th centuries [2]. With the arrival of the First Fleet in 1788 from England and subsequent establishment of settlements, chickens were introduced into the Island. There is now a feral chicken population that is believed to have originated from those early introductions, as a result of accidental and/or intentional release of European domestic breeds. However, there is ongoing debate about the genetic origins of those feral chickens because they could also have been introduced during the arrival of the Pitcairners and Melanesian students and/or during possible trading when ships en-route called in to the Island between the late 19th and early 20th Centuries [3,4] (Additional file 1: Figure S1A). In this context, the available DNA datasets of chickens provide a valuable resource for assessing the genetic background of those feral populations.

Ten divergent chicken mitochondrial DNA (mtDNA) haplogroups have been identified, with the majority of these exhibiting some form of restricted distribution on subcontinental scales [5-9]. Two of these haplogroups, E and D, are relevant to this study because they provide insights into the mtDNA signatures of Norfolk Island feral chickens. Haplogroup E is widespread among Indian, Middle Eastern and European chickens and is considered to be an indication that the roots of European chickens were in the Indian subcontinent. Haplogroup D (known also as haplogroup C [9,10]) is found in chickens from Japan, southeast China, India, Madagascar, Indonesia, the Philippines, Vietnam, Thailand, and Myanmar. Interestingly, this haplogroup contains a subgroup associated with contemporary chickens from the southeast Pacific and some ancient specimens from Easter Island [11], which might represent a genetic signature of an early chicken dispersal in the Pacific [9]. A detailed study of this subgroup indicated the presence of those ancient genetic signatures in contemporary chickens from Vanuatu and Guam [8]. Thus, haplogroup D is a useful source of information for assessing the contribution of Pacific chickens to specimens of unknown origin in the region.

Although the Norfolk Island feral chicken population is considered a threat to natural regeneration of native plant species and some endangered snails [12,13], these animals represent one of the few existing feral insular populations. This population provides an opportunity to investigate patterns of DNA variation in a population that has been isolated for the last 100–200 years. From a historical point of view, it is interesting to assess the genetic origins of Norfolk Island chickens because their genetic background is unknown and could be the result of possible multiple domestic chicken introductions. Here we attempt to resolve the genetic origins of Norfolk Island feral chickens by sequencing the mitochondrial control region. We estimated their phylogenetic relationships using a large dataset of approximately 3100 published sequences from around the world, including domestic chickens from mainland Australia.

## Methods

### Sample collection and DNA sequencing

Feral chickens were captured on Norfolk Island at eight sampling locations (Additional file 1: Figure S1B) using baited cages with a variety of colourful fruit, vegetables and bread. Blood samples were collected from the brachial vein of the wings from 27 chickens (Additional file 1: Table S1) and immediately transferred to FTA papers (Qiagen, Hilden, Germany) for subsequent DNA extraction using the QIAamp DNA Investigator Kit (Qiagen, Hilden, Germany). Additionally, we included 48 samples from Australian domestic chickens, which are known to have originated from European and Asian stocks and subsequently established and/or improved in Australia: Langshan Bantam (*n* = 4), Ancona Bantam (*n* = 2), Ancona (*n* = 2), Araucana (*n* = 2), Araucana Bantam (*n* = 1), Araucana Cross (*n* = 1), Australorp (*n* = 4), Barnevelder (*n* = 6), Hamburg (*n* = 1), Isa Brown (*n* = 2), Modern Game Bantam (*n* = 4), Rhode Island Red (*n* = 4), Sebright (*n* = 7), Spanish (*n* = 3), Sussex (*n* = 1), Welsummer (*n* = 2), Wyandotte Bantam (*n* = 1) and Wyandote (*n* = 1).

We chose to use the hypervariable region 1 (~540 bp) from the mitochondrial control region because it is highly polymorphic and informative for studying chicken populations, as demonstrated in a large number of previous studies [6-9,14-16]. This region was amplified using the following set of primers: 5’-AGGACTACGGCTTGAAAAGC-3’ and 5’-TGTGCCTGACCGAGGAACC AG-3’. DNA was amplified using the polymerase chain reaction (PCR) in 25 μl volumes containing 100–200 ng genomic DNA, 1× PCR buffer, 1.5 mM MgCl_2_, 0.12 mM dNTPs, 20 pmol of each primer, and 2 U *Taq* DNA polymerase (Promega, Madison, Wisconsin). PCR conditions included an initial denaturation at 94°C for 2 min, followed by 35 cycles of 25 s at 94°C, 35 s at 58°C, and 1 min 10 s at 72°C, and a final extension for 10 min at 72°C. Generation of the amplicons took place at the University of Sydney, while clean-up and Sanger sequencing were conducted at the Australian Genome Research Facility Ltd (Brisbane). Forward and reverse sequences were overlapped to obtain a consensus sequence of 540 bp for each sample, after excluding primer sequences. The haplotype sequences produced in this study were deposited into GenBank (accession numbers: KC347725–KC347735).

### Sequence data

We assembled two datasets in this study. The first dataset (488 bp) was used to assess the relationships of the Norfolk Island ferals and Australian domestic breeds to known chicken haplogroups. This dataset comprised 3063 sequences of domestic chickens and Red Junglefowl from Europe, Asia, Africa, Oceania, and the Americas, including the datasets used by Lee *et al. *[14], Gongora *et al*. [9], Kanginakudru *et al*. [5], and Berthouly-Salazar *et al. *[7]. The dataset also included more than 1700 chicken sequences (GenBank accession numbers GU447321–GU449100) analysed by Miao *et al. *[17]. Further details of the sources of these datasets are provided in Additional file 2: Table S2A.

Additional mtDNA sequences were available from published studies and/or GenBank but only overlapped between positions 167–367 of the mitochondrial genome [NC_001323.1]. To allow these sequences to be included in our analysis without introducing large amounts of missing data, we assembled a second, truncated dataset (200 bp) to estimate the relationships of the Norfolk Island chicken sequences within haplogroups E and D. This dataset comprised a total of 3223 sequences (Additional file 2: Table S2B), including 112 haplotypes from Island Southeast Asia and the Pacific [8,18], 10 sequences from Madagascar produced by Razafindraibe *et al. *[16], and 21 ancient DNA sequences from various locations in the Pacific (Thailand, Vanuatu, Niue, Solomon Islands, Hawaii, and Easter Island), South America (Chile, Bolivia, and Peru), and Spain [11,19,20]. Additional analyses confirmed that the truncated alignment was still informative (data not shown), with sufficient resolution to differentiate haplotypes and haplogroups.

For both datasets, sequences were aligned using MAFFT v.6 [21] based on the FFT-NS-1 strategy in which indels were excluded. To check the automated alignments, sequences were also aligned manually in BioEdit v. 7.1.3.0 [22]. FaBox 1.40 [23] was used to reduce the aligned datasets to unique haplotypes, which were then used to generate a multistate alignment of variable positions and to calculate haplotype frequencies (Additional file 3: Tables S3A, S3B and S3C).

### Phylogenetic analyses

Haplotypes were numbered by FaBox when collapsed. Neighbour-joining analysis was performed using MEGA 5 [24], based on the Kimura-2-parameter model. Median networks were estimated using the median-joining algorithm in Network 4.6.1.0 [25], with default settings and star contraction options (with a threshold connection limit of five) to collapse very closely related sequences into haplotypes. To produce a simplified view of the relationships among Norfolk Island feral and Australian chickens, an additional median-joining network analysis was performed on the most frequent haplotype from each major haplogroup identified in the 200 bp dataset. To improve visualisation of the relationships among haplotypes, a subset of the 200 bp dataset corresponding to haplogroups D and E was used for neighbour-joining and median-joining network analyses using the settings described above.

Bayesian phylogenetic analyses were also performed for haplogroups E and D using MrBayes 3 [26]. Sequence AF512265, belonging to the main haplotype within haplogroup E, was used as an outgroup for the analysis of haplogroup D while sequence AF512158, belonging to the main haplotype within haplogroup D, was used as an outgroup for the analysis of haplogroup E. The best-fitting substitution model was selected using jModelTest 2 [27,28] according to the Akaike information criterion [29]. Posterior estimates of parameters, including the tree topology, were obtained using Markov chain Monte Carlo sampling. Samples were drawn every 100 steps over a total of 1.5 million steps for haplogroup E and 6 million steps for haplogroup D, with the first 25% of steps discarded as burn-in. Two independent runs were performed, each with one cold and three heated chains. Sufficient sampling from the stationary distribution was checked by inspecting the standard deviation of split frequencies.

## Results and discussion

### Diversity and distribution of haplotypes in haplogroups D and E

**Figure 1:**
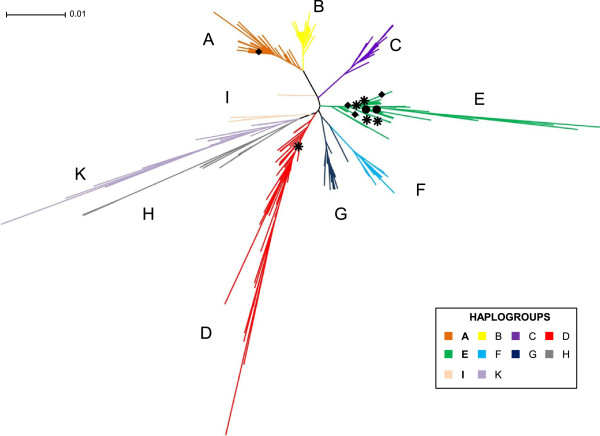
**Neighbour-joining tree showing the relationships of mitochondrial DNA (488 bp) from worldwide, Norfolk Island, and mainland Australia chickens.** This represents 425 haplotypes from 3063 sequences (Additional file 3: Table S3A). Haplotypes are classified into ten haplogroups (A-I, K). Symbols represent the following: (*) common haplotypes among Norfolk Island ferals and Australian domestic chickens; (●) haplotypes present only in Norfolk Island ferals; and (♦) haplotypes present only in Australian domestic chickens. Major haplogroups are represented by different colours as described in the legend.

Median networks and neighbour-joining analyses revealed ten haplogroups/clades (A-I and K) defined previously by Liu *et al. *[6] and Berthouly *et al. *[7], respectively (Figure 1). Most haplogroups included both Red Junglefowl and domestic chickens, with the exception of haplogroups C and K which consist exclusively of either domestic chicken or Red Junglefowl haplotypes (Figure 1; Additional file 3). Of relevance to this study, haplogroup E appears to be widespread in specimens from Europe, western Asia, the Indian subcontinent, the Pacific, and South America (Additional file 4: Figure S4A). In contrast, haplogroup D appears to be more restricted to the Indian subcontinent, Japan, Madagascar, across China (except in the north) and mainland and Island Southeast Asia including Indonesia and other countries in the Pacific (Additional file 4: Figure S4A).

**Figure 2:**
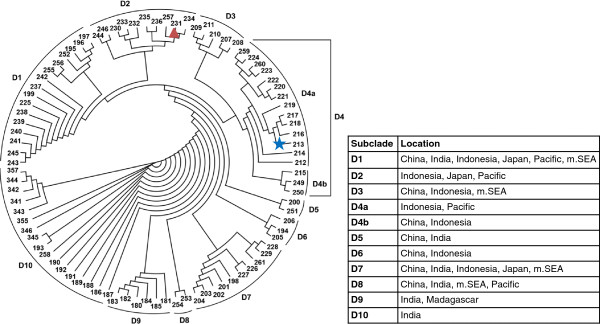
**Neighbour-joining tree showing the relationships of mitochondrial DNA (200 bp) of chickens within haplogroup D.** The geographical distribution of the subclades is presented in Figure 3. The term 'Pacific’ refers to the geographical region including Easter Island, Guam, Hawaii, the Philippines and Vanuatu, while mainland Southeast Asia (m.SEA) consists of Laos, Malaysia, Myanmar, Thailand and Vietnam. The coloured star highlights haplotype h213 in which ten Norfolk Island feral chickens and three Australian domestic chickens cluster. The red triangle indicates haplotype h231, which constitutes an ancient genetic signature of the Pacific by clustering ancient Easter Island and Hawaiian samples with Vanuatu chickens.

**Figure 3:**
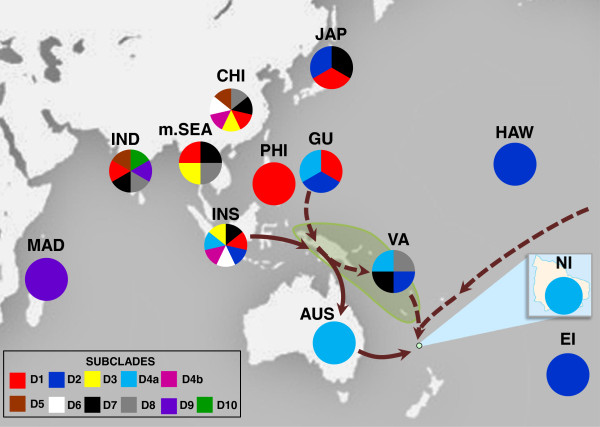
**Geographical distribution of haplogroup D based on 200 bp from the mitochondrial control region**. Pies define the presence of each subclade from Figure 2 according to locations: AUS, Australia; CHI, China; EI, Easter Island; GU, Guam; HAW, Hawaii; IND, India; INS, Indonesia; JAP, Japan; MAD, Madagascar; NI, Norfolk Island; PHI, Philippines; m.SEA, mainland Southeast Asia (including Laos, Myanmar, Thailand, and Vietnam); and VA, Vanuatu. The green shaded shape represents Melanesia, while the two arrow types present the two possible routes for the introduction of haplogroup D to Norfolk Island: the solid arrow represents a possible introduction via Australia; and the two dashed arrows represent an introduction directly from Southeast Asia or the Pacific.

Median networks estimated from the 200 bp dataset show that haplogroup D consists of 88 haplotypes, which is greater than that observed for other haplogroups (7–54 haplotypes). Haplogroup D represents approximately 24.6% of the known global haplotype diversity in chicken mtDNA, with Indonesia alone contributing 48.8% of this. On a finer scale, haplogroup D appears to show some subdivision at the geographical level (Additional file 5: Figure S5A), which is supported by neighbour-joining analyses (Figure 2). However, our Bayesian analysis does not provide sufficient phylogenetic resolution for those clades (Additional file 5: Figure S5B). The neighbour-joining tree shows that while some subclades (D1, D3, D7, D8-D9) consist of haplotypes from both mainland Asia and Island Southeast Asia or Madagascar, others (D4b, D6) appear to be restricted to Indonesia and China (Figure 3) or to be separate from the others (such as D10, related to Indian chicken sequences). Although bootstrap support is low for these subclades, 78 of the 88 haplotypes from haplogroup D, including 34 of those from Indonesia, appear to be restricted to individual countries across its geographical distribution (Figure 3). Of relevance to the interpretation of our results is subclade D4a, which consists of chickens from Indonesia, Guam, and Vanuatu (Figure 3). Given the wide distribution of some of the D haplotypes in the Indian subcontinent and western Asia, the restricted distribution of others in Island Southeast Asian and Pacific regions provides additional mtDNA signatures for assessing the origins of chickens such as the Norfolk Island ferals.

Haplogroup/clade E consists of 54 haplotypes that constitute approximately 15% of the worldwide diversity. Neighbour-joining tree and median networks show some structure but no specific phylogeographical pattern. For instance, subclades from the neighbour-joining tree, E1 and E2 represented by haplotypes h130-h155, h157, h165-h166, h75, h336, and h358, have a worldwide distribution. In contrast, subclades E3 – E5, represented by haplotypes h156-h164, h166-h173, h328, h337-h340, and h350, appear to be restricted to the Indian subcontinent and China, with the exception of haplotype h157 which is also present in Japan and western Asia (Additional file 6: Figure S6B). Our analyses also show that haplotypes h130, h131, and h146 have the widest distribution whereas h145 appears to be more localised to the Indian subcontinent and western Asia.

During the course of our study, Miao *et al. *[17] independently and similarly found a similar geographical distribution of haplogroup E and D, including some with widespread and some with restricted haplotype distributions in South and Southeast Asia. However, their nomenclature is different from that used in our study. This is because we included additional sequences, particularly from Indonesia, to provide a finer-scale phylogeographical analysis of those haplogroups and thus defined subclades not detected in that study.

### Genetic relationships of Norfolk Island feral chickens

Seven control region haplotypes (h130, h131, h133, h143, h145, h146 and h213) were found among the Norfolk Island feral chickens (Table 1; Additional file 1: Table S1). Five of these haplotypes (h130, h 131, h145, h146, and h213) were shared between Norfolk Island feral and Australian mainland domestic chickens.

**Table 1:** Frequency of haplotypes present in Australian and Norfolk Island feral chickens

**Location**	**Haplogroup**											**Total**
	**A**	**D**	**E**									
	**h1**	**h213**	**h130**	**h131**	**h133**	**h143**	**h145**	**h146**	**h153**	**h157**	**h166**	
Australia	5	3	4	18			14	1	1	1	1	48
Norfolk Island		10	5	7	2	1	1	1				27

Six of the Norfolk Island feral chicken haplotypes (h130, h131, h133, h143, h145, and h146) fall within haplogroup E (Additional file 3: Table S3B; Additional file 4: Figures S4C). Most of these haplotypes are known to have widespread distributions, including h131 (known as haplotype *E1*) which represents the most common chicken haplotype found across the world [6,9]. Haplotype h143 is an exception because it is placed separately from the other E subclades (Additional file 6: Figure S6B). The wide global distribution of the E haplotypes in Norfolk Island feral chickens suggests that they had a very efficient agent of dispersal, such as the navy of the greatest colonising power in the 18th Century (i.e., the British). This proposition is plausible, especially given that the global distribution of the group E haplotypes matches those countries that were British colonies or known ports of call [30,31]). It is known that some livestock was added during the stops that the First Fleet made in Cape Town, a Dutch colony at that time, and/or in Rio de Janeiro, a Portuguese colony at the time [32-34]. In addition, it is recorded that a First Fleet ship went to China in 1790, where those haplotypes E are also found, to bring livestock to the Island [35].

The phylogenetic position of h143 is defined as an intermediate haplotype connecting the most common haplotype worldwide, h131, to a branch of Asian (Indian/Chinese) haplotypes E consisting of haplotypes h167-h173 (Additional file 6: Figure S6A). The unique polymorphism that differentiates h143 from h131 does not necessarily indicate a new mutation that arose on the Norfolk Island population; it could have occurred elsewhere in continental Asia. The identification of this novel haplotype provides a potential genetic resource to trace the origins of haplogroup E in these ferals when additional European or Asian chicken data become available.

Haplotype h213, belonging to subclade D4a, was found in 10 of the Norfolk Island ferals and in three Australian domestic chicken breeds (Australorp, Sussex, and Araucana) (Table 1; Additional file 1: Table S1). The interpretation of the presence of this haplotype in the Norfolk Island feral chickens is challenging because of the limited records of chicken introductions. Nevertheless, several scenarios can be considered. This haplotype could have been the result of indirect introgression of Asian domestic genes introduced into Australia and subsequently to Norfolk Island via Europeans. An alternative source of haplotype h213 could have been the trade from ships that were en-route from Tahiti, Fiji, New Zealand, and the 'South Seas’ (as well as Peru and Chile) and called in to the Island between 1801 and 1813 [4]. However, h213 or very closely related haplotypes have not been found in some of those countries.

Although ancient genetic signatures of Pacific chicken dispersal [8,9] have not been specifically associated to h213, this haplotype is found in chickens from countries that have been suggested to show such signatures (h231, subclade D2)(Figure 2). However, these haplotypes are not closely related (Figure 2 and Additional file 5: Figure S5A) and there is no archaeological evidence of chickens in Polynesian sites in Norfolk Island [36] that supports pre-European introductions, so the link to those ancient genetic signatures is uncertain.

We suggest that the most probable primary origin of haplotype h213 in Norfolk Island feral chickens is in Island Southeast Asia or the Pacific, because of its restricted distribution in this region (Figure 3). We propose at least two routes of introduction: i) directly from Australia after introgression of Island Southeast Asia or Pacific genes; ii) from the Pacific as a result of the settlement of Pitcairners [37,38] and/or Melanesians [37] on Norfolk Island during the British administration in the 19th Century. Thus, our results suggest that an important part of the origins of the Norfolk Island feral chickens fall somewhere in Island Southeast Asia and/or Australia, which is consistent with the geographical proximity of these regions. It is difficult to define exactly when and how these introductions occurred, because there have probably been multiple introductions from different geographical regions. However, given that Australian domestic and Norfolk Island feral chickens share a majority of haplotypes and considering that Australia has been a major departure point of ships arriving at Norfolk Island, Australian breeds have probably contributed substantially to the genetic diversity of that feral population.

Tracing the specific origin of Norfolk Island feral chickens was not fully resolved in the present study. This is a consequence of the complex history of global chicken dispersal, which has led to a remarkable lack of phylogeographic information in the mtDNA. DNA extraction and analyses of chicken bones found in archaeological remains dating from early British and subsequent settlements on the Island may shed light on the founders of the Norfolk Island feral chicken population. This has the potential to open new research in molecular ecology to understand whether the microenvironment of Norfolk Island influenced the legacy of E and/or D haplotypes, as European and Pacific chickens may have responded differently to the warm humid temperate conditions found there. Given the presence of other feral chicken populations, including those from the Cocos Islands in the Indian Ocean and from Kauai Island in Hawaii, the phylogenetic framework presented here might be useful for investigating their genetic origins and patterns of DNA sequence variation between populations.

## Conclusions

We have provided the first genetic assessment of the Norfolk Island feral chickens and have identified that two possible mtDNA sources (haplogroups D and E) have contributed to their genetic make-up. We suggest that the proposed European origin of that population requires further investigation, while providing evidence that Island Southeast Asia/Pacific chickens have made a genetic contribution to this feral population. Furthermore, we found a discrete geographical distribution of haplotypes in Island Southeast Asia and the Pacific region, which provides a useful genetic signature for assessing the contribution of chickens from this region to specimens of unknown origin. Further studies, particularly focusing on chickens from Southeast Asia, the Pacific, and Australasia, could evaluate the diversity of coding genes associated with particular phenotypic traits (e.g. skin, plumage and eggshell colour and features of the comb and wattles). In addition, genome-wide SNP analyses can be used to provide a more comprehensive understanding of the genetic diversity and origins of different populations. In view of the worldwide interest in the characterisation and conservation of poultry resources and their role in emerging diseases, it is important to investigate the genomic diversity and immunological fitness of chickens from Indonesia and the Pacific, including Norfolk Island.

## Abbreviations

mtDNA: Mitochondrial DNA; NI: Norfolk Island.

## Competing interests

The authors declare that they have no competing interests.

## Authors’ contributions

SLS sampled the feral chicken population, extracted the DNA and prepared a preliminary draft of the manuscript; SK undertook most of the data analyses, contributed to the preparation of the main body of the manuscript, and prepared the supplementary material; BB initiated this work, participated in its design, contributed to interpretation of sampling and data from a historical perspective, and contributed to the preparation of the manuscript; SYWH supervised some of the analyses and contributed to the preparation of the manuscript; JG designed and directed this study and contributed substantially to the analyses, interpretation of data and preparation of the manuscript and supervised SLS. All authors read and approved the final manuscript.

## Supplementary Material

Additional file 1: Figure S1A Timeline of human presence on Norfolk Island. The timeline shows settlements on the Island from the first arrival of Polynesians in the 13th Century to the most recent settlement by Europeans and Pitcairners. The historical record of chicken introduction is in red, with uncertainty indicated with question marks. **Figure S1B.** Map of Norfolk Island indicating the sampling sites. **Table S1.** Voucher information for Norfolk Island and Australian samples. Click here for file

Additional file 2: Table S2A List of sequences used in the 488 bp dataset. **Table S2B.** List of sequences used in the 200 bp dataset. Click here for file

Additional file 3: Table S3A Details for the 425 haplotypes generated by the 488 bp dataset. The table presents the frequencies of the 425 haplotypes generated by the 488 bp dataset, the sequences that belong to each haplotype and their geographical origin, as well as the haplogroup in which haplotypes cluster. The clustering of Red Junglefowl in the haplotypes is indicated with √ and Red Junglefowl sequences are highlighted in red. The table also defines the specific region of China to which sequences belong and the corresponding haplotype number for each haplotype in the 200 bp dataset. **Table S3B.** Details for the 358 haplotypes generated by the 200 bp dataset. Table presenting the frequencies of the 358 haplotypes generated by the 200 bp dataset, the sequences that belong to each haplotype and their geographical origin, as well as the haplogroup in which haplotypes are clustered. Sequences representing ancient samples are denoted with ♦. The table also defines the specific region of China to which sequences belong. **Table S3C.** Polymorphic sites of the haplotypes generated by the 488 bp. The polymorphic sites that are included in the 200 bp dataset are highlighted with a shaded colour. Haplotypes are grouped according to haplogroup. Click here for file

Additional file 4: Figure S4A Frequency of haplogroups by geographical location based on the 200 bp of the 3223 mtDNA sequences. Sampling locations followed by sampling size: AUS, Australia-48; BOL, Bolivia-2; CH, China (C.CH, Central China-415; E.CH, Eastern China-172; N.CH, Northern China-108; S.CH, Southern China-1076; W. CH, Western China-239); CHL, Chile-42; EI, Easter Island-2; EU, Europe-60; GU, Guam-5; HAW, Hawaii-7; IND, India-329; INS, Indonesia-94; JAP, Japan-152; KOR, Korea-31; MD, Madagascar-11; NI, Norfolk Island-27; NU, Niue-1; PER, Peru-1; PHI, Philippines-1; m.SEA, mainland Southeast Asia (including Laos, Malaysia, Myanmar, Thailand, and Vietnam)-292; SOL, Solomon Islands-3; VA, Vanuatu-41; WA, Western Asia-16. **Figure S4B.** Median networks with star contraction of all haplotypes for the 200 bp segment of the mitochondrial control region. Nodes are coloured according to haplogroup. Median vectors are shown in black. Better resolution of haplogroups E and D is provided in Additional files 5: Figure S5A and Additional file 6: Figure S6A. **Figure S4C.** Median network showing the relationships among Australian and Norfolk Island haplotypes, produced by the 200 bp segment of the mitochondrial control region, with the most frequent haplotype of the remaining haplogroups. Norfolk Island and Australian haplotypes are clustered within the dashed oval. Circle size is proportional to haplotype frequency (Additional file 3, Table S3B). Slashes (//) indicate partial omission of branch length due to improved clarity, with the number of mutations shown next to the slashes. Nodes are coloured according to haplogroup, as shown in the legend. Click here for file

Additional file 5: Figure S5A Median network showing the relationships between haplotypes within haplogroup D in the 200 bp enriched dataset. Haplotype numbers are shown next to nodes. Node size is proportional to the frequency of the corresponding haplotypes, as shown in the circles, with numbers on the right. Branch lengths have been modified for visual clarity. The geographical location of samples is given in colour, as indicated in the legend. Dashed shapes refer to nine subclades, as defined in Figures 2 and 3. The red dashed shape highlights subclade D4a, where Norfolk Island and Australian sequences are placed. **Figure S5B.** Bayesian phylogenetic tree for haplogroup D based on the 200 bp enriched dataset. Numbers on clades show posterior probabilities. Sequence AF512265 belongs to haplotype 131, the main haplotype within haplogroup E, and was used as an outgroup. Click here for file

Additional file 6: Figure S6A Median network showing the relationships among haplotypes within haplogroup E in the 200 bp enriched dataset. Haplotype numbers are shown next to nodes and node size is proportional to the frequency of the corresponding haplotypes, as shown in the circles with numbers on the right. Branch lengths are proportional to mutations, except for the branch leading from median vector (mv) 14 to haplotype 337 (8 mutations) and the branch leading from median vector 15 to haplotype 350 (14 mutations). Red highlighting shows the branches that connect the unique in Norfolk Island haplotype h143 with the most frequent haplotype worldwide, h131, and the haplotype h168 that is present in China and India. Only one mutation separates haplotype h143 from h131, while two mutations arose between this haplotype and the Chinese/Indian branch (h168). **Figure S6B.** Neighbour-joining tree (K2P) for haplotypes within haplogroup E based on the 200 bp enriched dataset. Coloured boxes next to node names define the location of each haplotype. The world maps next to haplotypes 130, 131, and 146 indicate the widespread nature of their distribution, while haplotypes 143 and 145 are circled. Haplotype 143 is restricted to Norfolk Island feral chickens. Haplotype 145, which is present in China, Japan, India, Guam, and Australia, shows a narrower distribution than the rest of the Norfolk Island haplotypes. **Figure S6C.** Bayesian phylogenetic tree for haplogroup E based on the 200 bp enriched dataset. Numbers on clades show posterior probabilities. Sequence AF512158 which belongs to haplotype 253, the main haplotype within haplogroup D, was used as an outgroup. The asterisk (*) indicates the haplotypes to which Norfolk Island chickens belong. Click here for file
