# Supplementary material for: Australian and Pacific contributions to the genetic diversity of Norfolk Island feral chickens
Source: BMC Genet. 2013 Sep 24;14:91. doi: 10.1186/1471-2156-14-91 (PMC3850513; doi:10.1186/1471-2156-14-91)
Supplement: Additional file 1: Figure S1A — Timeline of human presence on Norfolk Island. The timeline shows settlements on the Island from the first arrival of Polynesians in the 13th Century to the most recent settlement by Europeans and Pitcairners. The historical record of chicken introduction is in red, with uncertainty indicated with question marks. Figure S1B. Map of Norfolk Island indicating the sampling sites. Table S1. Voucher information for Norfolk Island and Australian samples. [file 1471-2156-14-91-S1.doc]

**Additional file 1**


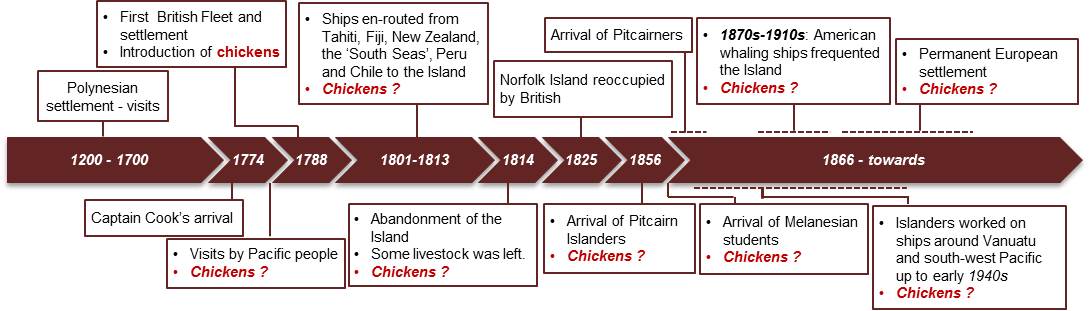


**Figure S1A:** Timeline of human presence on Norfolk Island. The timeline shows settlements on the Island from the first arrival of Polynesians in the 13^th^ Century to the most recent settlement by Europeans and Pitcairners. The historical record of chicken introduction is in red, with uncertainty indicated with question marks.


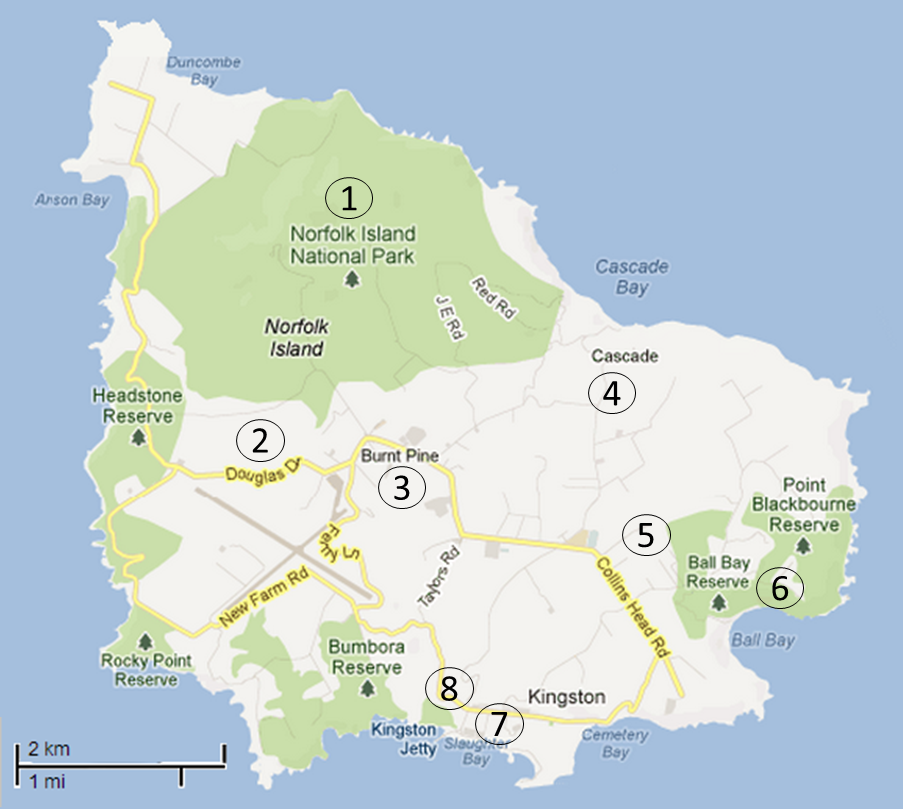


**Figure S1B:** Map of Norfolk Island. Numbers indicate the eight sampling sites.

**Table S1:** Voucher information for Norfolk Island and Australian chicken samples.

| **Sample ID** | **Breed or type** | **Norfolk Island/Australian haplotype name in the 200bp dataset** | **Haplotype No. as indicated in network analyses of the 200bp dataset** |
| --- | --- | --- | --- |
| ALB1 | Australian Langshan Bantam | h131 | 131 |
| ALB2 | Australian Langshan Bantam | h131 | 131 |
| ALB3 | Australian Langshan Bantam | h131 | 131 |
| ALB4 | Australian Langshan Bantam | h131 | 131 |
| AnB1 | Ancona Bantam | h131 | 131 |
| AnB2 | Ancona Bantam | h131 | 131 |
| Anc1 | Ancona | h131 | 131 |
| Anc2 | Ancona | h131 | 131 |
| ARA1 | Araucana | h213 | 213 |
| ARA2 | Araucana | h145 | 145 |
| ARAb | Araucana Bantam | h131 | 131 |
| ARAX | Araucana Cross | h130 | 130 |
| Aus1 | Australorp | h131 | 131 |
| Aus2 | Australorp | h130 | 130 |
| Aus3a | Australorp | h131 | 131 |
| Aus4 | Australorp | h213 | 213 |
| Barn1 | Barnevelder | h145 | 145 |
| Barn2 | Barnevelder | h145 | 145 |
| Barn3 | Barnevelder | h145 | 145 |
| Barn4 | Barnevelder | h145 | 145 |
| Barn5 | Barnevelder | h145 | 145 |
| Barn10 | Barnevelder | h145 | 145 |
| HAM | Hamburg | h166 | 166 |
| ISA1 | Isa Brown | h131 | 131 |
| ISA2 | Isa Brown | h146 | 146 |
| MGB1 | Modern game Bantam | h145 | 145 |
| MGB2 | Modern game Bantam | h145 | 145 |
| MGB3 | Modern game Bantam | h131 | 131 |
| MGB4 | Modern game Bantam | h145 | 145 |
| RIRB1 | Rhode Island Red | h1 | 1 |
| RIRB2 | Rhode Island Red | h1 | 1 |
| RIRB3 | Rhode Island Red | h1 | 1 |
| RIRB4 | Rhode Island Red | h1 | 1 |
| SEB1 | Sebright | h145 | 145 |
| SEB2 | Sebright | h130 | 130 |
| SEB3 | Sebright | h145 | 145 |
| SEB4 | Sebright | h145 | 145 |
| SEB5 | Sebright | h145 | 145 |
| SEB6 | Sebright | h157 | 157 |
| SEB7 | Sebright | h130 | 130 |
| Sp1 | Spanish | h131 | 131 |
| Sp2 | Spanish | h131 | 131 |
| Sp3 | Spanish | h153 | 153 |
| SUS | Sussex | h213 | 213 |
| WEL1 | Welsummer | h131 | 131 |
| WEL2 | Welsummer | h131 | 131 |
| WYAB | Wyandotte Bantam | h1 | 1 |
| WYAS | Wyandotte | h131 | 131 |
| NI1 | Feral | h213 | 213 |
| NI2a | Feral | h143 | 143 |
| NI3 | Feral | h131 | 131 |
| NI4a | Feral | h131 | 131 |
| NI5a | Feral | h131 | 131 |
| NI6 | Feral | h145 | 145 |
| NI7 | Feral | h131 | 131 |
| NI8 | Feral | h133 | 133 |
| NI9 | Feral | h130 | 130 |
| NI10 | Feral | h213 | 213 |
| NI11 | Feral | h130 | 130 |
| NI12 | Feral | h130 | 130 |
| NI13 | Feral | h130 | 130 |
| NI14 | Feral | h146 | 146 |
| NI15 | Feral | h133 | 133 |
| NI16 | Feral | h213 | 213 |
| NI17 | Feral | h130 | 130 |
| NI18 | Feral | h213 | 213 |
| NI19 | Feral | h131 | 131 |
| NI20 | Feral | h131 | 131 |
| NI21 | Feral | h131 | 131 |
| NI22 | Feral | h213 | 213 |
| NI23 | Feral | h213 | 213 |
| NI24 | Feral | h213 | 213 |
| NI25 | Feral | h213 | 213 |
| NI26a | Feral | h213 | 213 |
| NI27 | Feral | h213 | 213 |
