# Supplementary material for: Australian and Pacific contributions to the genetic diversity of Norfolk Island feral chickens
Source: BMC Genet. 2013 Sep 24;14:91. doi: 10.1186/1471-2156-14-91 (PMC3850513; doi:10.1186/1471-2156-14-91)
Supplement: Additional file 4: Figure S4A — Frequency of haplogroups by geographical location based on the 200 bp of the 3223 mtDNA sequences. Sampling locations followed by sampling size: AUS, Australia-48; BOL, Bolivia-2; CH, China (C.CH, Central China-415; E.CH, Eastern China-172; N.CH, Northern China-108; S.CH, Southern China-1076; W. CH, Western China-239); CHL, Chile-42; EI, Easter Island-2; EU, Europe-60; GU, Guam-5; HAW, Hawaii-7; IND, India-329; INS, Indonesia-94; JAP, Japan-152; KOR, Korea-31; MD, Madagascar-11; NI, Norfolk Island-27; NU, Niue-1; PER, Peru-1; PHI, Philippines-1; m.SEA, mainland Southeast Asia (including Laos, Malaysia, Myanmar, Thailand, and Vietnam)-292; SOL, Solomon Islands-3; VA, Vanuatu-41; WA, Western Asia-16. Figure S4B. Median networks with star contraction of all haplotypes for the 200 bp segment of the mitochondrial control region. Nodes are coloured according to haplogroup. Median vectors are shown in black. Better resolution of haplogroups E and D is provided in Additional files 5: Figure S5A and Additional file 6: Figure S6A. Figure S4C. Median network showing the relationships among Australian and Norfolk Island haplotypes, produced by the 200 bp segment of the mitochondrial control region, with the most frequent haplotype of the remaining haplogroups. Norfolk Island and Australian haplotypes are clustered within the dashed oval. Circle size is proportional to haplotype frequency (Additional file 3, Table S3B). Slashes (//) indicate partial omission of branch length due to improved clarity, with the number of mutations shown next to the slashes. Nodes are coloured according to haplogroup, as shown in the legend. [file 1471-2156-14-91-S4.doc]

**Additional file 4**

**
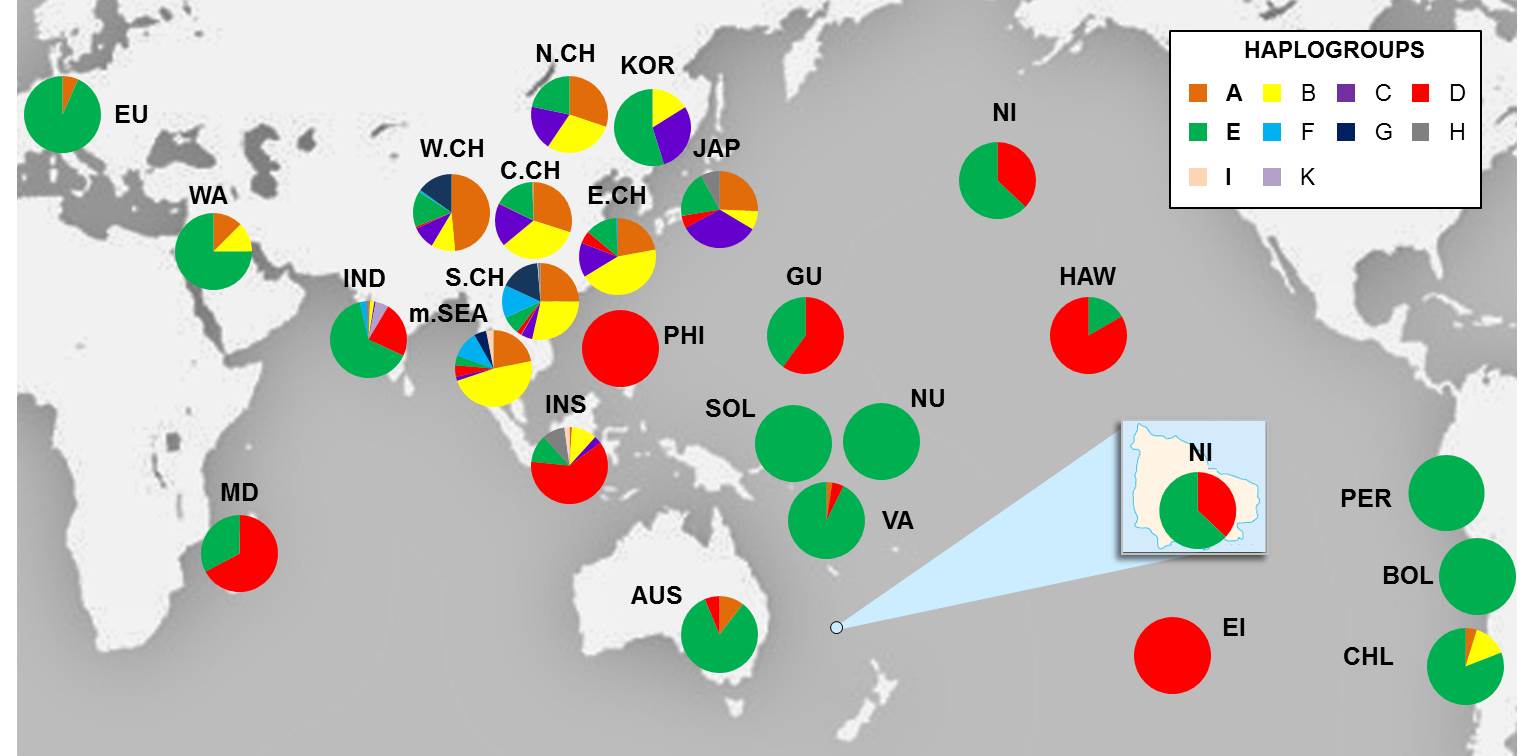
**

**Figure S4A:** Frequency of haplogroups by geographical location based on the 200 bp of the 3223 mtDNA sequences. Sampling locations followed by sampling size: AUS, Australia-48; BOL, Bolivia-2; CH, China (C.CH, Central China-415; E.CH, Eastern China-172; N.CH, Northern China-108; S.CH, Southern China-1076; W. CH, Western China-239); CHL, Chile-42; EI, Easter Island-2; EU, Europe-60; GU, Guam-5; HAW, Hawaii-7; IND, India-329; INS, Indonesia-94; JAP, Japan-152; KOR, Korea-31; MD, Madagascar-11; NI, Norfolk Island-27; NU, Niue-1; PER, Peru-1 ; PHI, Philippines-1; m.SEA, mainland Southeast Asia (including Laos, Malaysia, Myanmar, Thailand, and Vietnam)-292; SOL, Solomon Islands-3; VA, Vanuatu-41; WA, Western Asia-16.

**
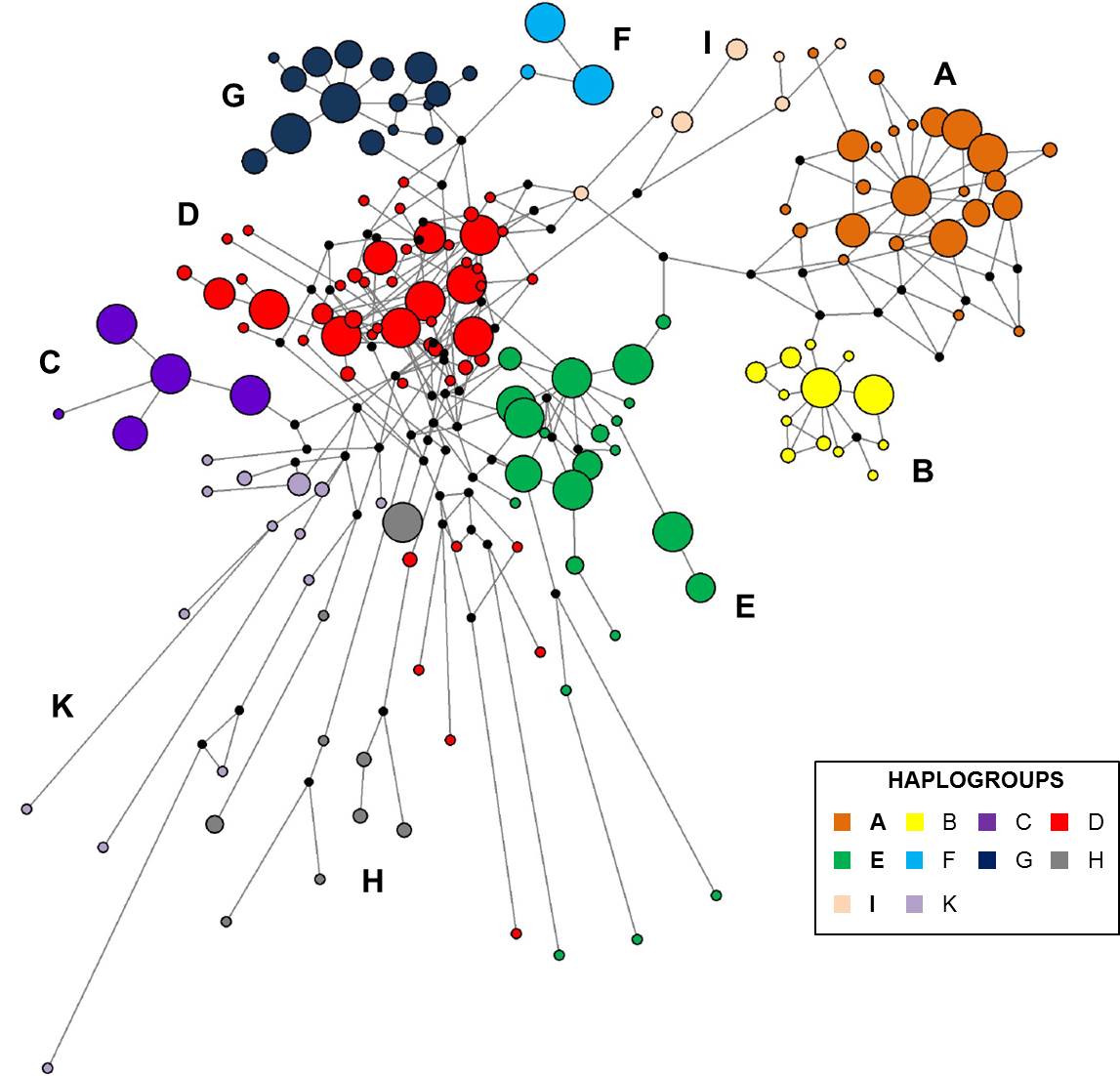
**

**Figure S4B:** Median networks with star contraction of all haplotypes for the 200 bp segment of the mitochondrial control region. Nodes are coloured according to haplogroup. Median vectors are shown in black. Better resolution of haplogroups E and D is provided in Additional files 5 (Figure S5A) and Additional file 6 (Figure S6A).


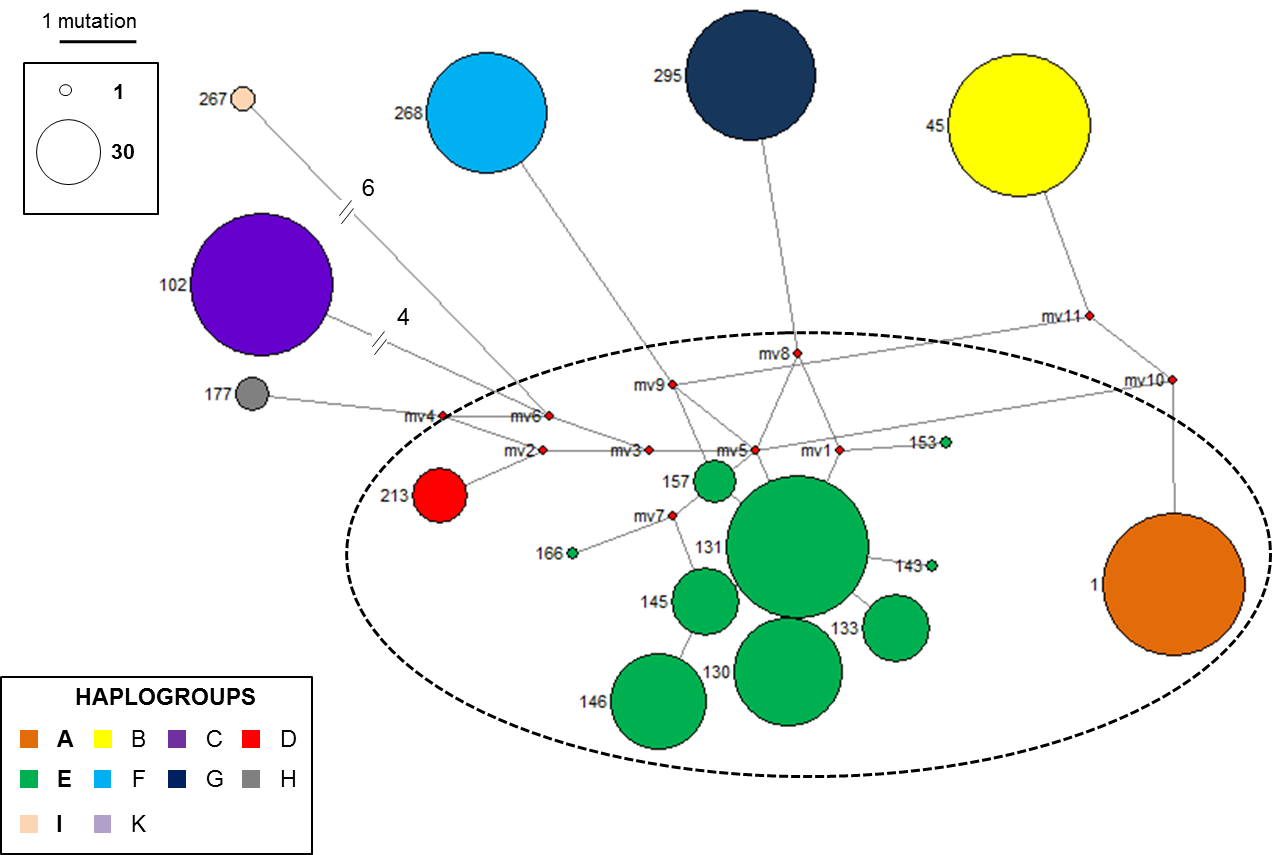


**Figure S4C:** Median network showing the relationships among Australian and Norfolk Island haplotypes, produced by the 200 bp segment of the mitochondrial control region, with the most frequent haplotype of the remaining haplogroups. Norfolk Island and Australian haplotypes are clustered within the dashed oval. Circle size is proportional to haplotype frequency (Additional file 3, Table S3B). Slashes (//) indicate partial omission of branch length due to improve clarity, with the number of mutations shown next to the slashes. Nodes are coloured according to haplogroup, as shown in the legend.
