# Supplementary material for: Australian and Pacific contributions to the genetic diversity of Norfolk Island feral chickens
Source: BMC Genet. 2013 Sep 24;14:91. doi: 10.1186/1471-2156-14-91 (PMC3850513; doi:10.1186/1471-2156-14-91)
Supplement: Additional file 5: Figure S5A — Median network showing the relationships between haplotypes within haplogroup D in the 200 bp enriched dataset. Haplotype numbers are shown next to nodes. Node size is proportional to the frequency of the corresponding haplotypes, as shown in the circles, with numbers on the right. Branch lengths have been modified for visual clarity. The geographical location of samples is given in colour, as indicated in the legend. Dashed shapes refer to nine subclades, as defined in Figures 2 and 3. The red dashed shape highlights subclade D4a, where Norfolk Island and Australian sequences are placed. Figure S5B. Bayesian phylogenetic tree for haplogroup D based on the 200 bp enriched dataset. Numbers on clades show posterior probabilities. Sequence AF512265 belongs to haplotype 131, the main haplotype within haplogroup E, and was used as an outgroup. [file 1471-2156-14-91-S5.doc]

**Additional file 5**

**
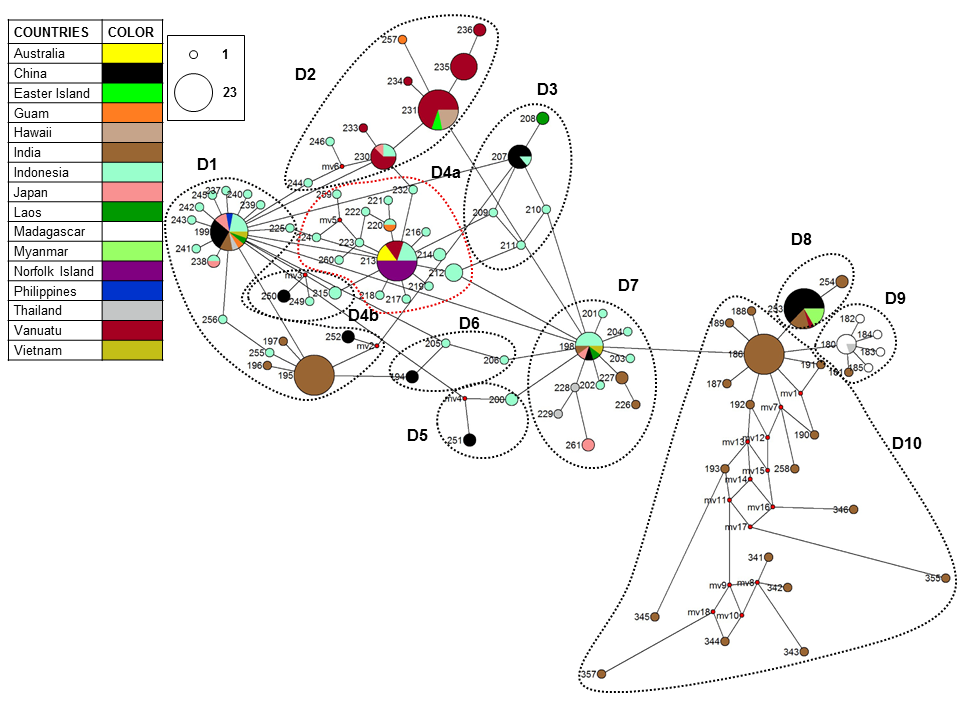
**

**Figure S5A:** Median network showing the relationships of haplotypes within haplogroup D in the 200 bp enriched dataset. Haplotype numbers are shown next to nodes. Node size is proportional to the frequency of the corresponding haplotypes, as shown in the circles with numbers on the right. Branch lengths have been modified for visual clarity. The geographical location of samples is given in colour, as indicated in the legend. Dashed shapes refer to nine subclades, as defined in Figures 2 and 3. The red dashed shape highlights subclade D4a, where Norfolk Island and Australian sequences are placed.


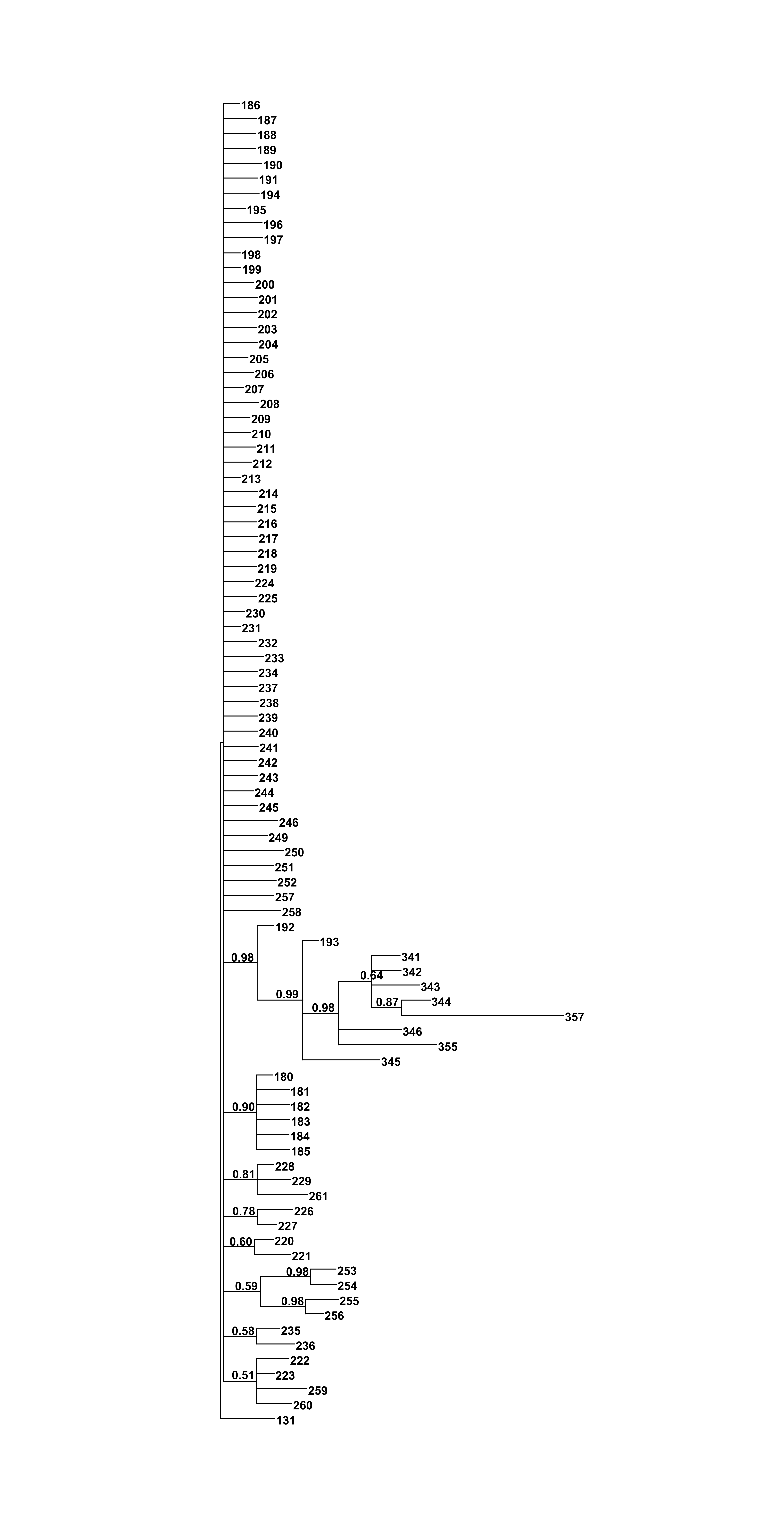


**Figure S5B:** Bayesian phylogenetic tree for haplogroup D based on the 200 bp enriched dataset. Numbers on clades show posterior probabilities. The sequence AF512265, belonging to haplotype 131, the main haplotype within haplogroup E, was used as an outgroup.
