# Supplementary material for: Australian and Pacific contributions to the genetic diversity of Norfolk Island feral chickens
Source: BMC Genet. 2013 Sep 24;14:91. doi: 10.1186/1471-2156-14-91 (PMC3850513; doi:10.1186/1471-2156-14-91)
Supplement: Additional file 6: Figure S6A — Median network showing the relationships among haplotypes within haplogroup E in the 200 bp enriched dataset. Haplotype numbers are shown next to nodes and node size is proportional to the frequency of the corresponding haplotypes, as shown in the circles with numbers on the right. Branch lengths are proportional to mutations, except for the branch leading from median vector (mv) 14 to haplotype 337 (8 mutations) and the branch leading from median vector 15 to haplotype 350 (14 mutations). Red highlighting shows the branches that connect the unique in Norfolk Island haplotype h143 with the most frequent haplotype worldwide, h131, and the haplotype h168 that is present in China and India. Only one mutation separates haplotype h143 from h131, while two mutations arose between this haplotype and the Chinese/Indian branch (h168). Figure S6B. Neighbour-joining tree (K2P) for haplotypes within haplogroup E based on the 200 bp enriched dataset. Coloured boxes next to node names define the location of each haplotype. The world maps next to haplotypes 130, 131, and 146 indicate the widespread nature of their distribution, while haplotypes 143 and 145 are circled. Haplotype 143 is restricted to Norfolk Island feral chickens. Haplotype 145, which is present in China, Japan, India, Guam, and Australia, shows a narrower distribution than the rest of the Norfolk Island haplotypes. Figure S6C. Bayesian phylogenetic tree for haplogroup E based on the 200 bp enriched dataset. Numbers on clades show posterior probabilities. Sequence AF512158 which belongs to haplotype 253, the main haplotype within haplogroup D, was used as an outgroup. The asterisk (*) indicates the haplotypes to which Norfolk Island chickens belong. [file 1471-2156-14-91-S6.doc]

**Additional file 6**

**
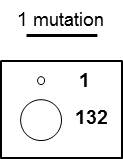
**

//

//

**Figure S6A:** Median network showing the relationships among haplotypes within haplogroup E in the 200 bp enriched dataset. Haplotype numbers are shown next to nodes and node size is proportional to the frequency of the corresponding haplotypes, as shown in the circles with numbers on the right. Branch lengths are proportional to mutations, except for the branch leading from median vector (mv) 14 to haplotype 337 (8 mutations) and the branch leading from median vector 15 to haplotype 350 (14 mutations). Red highlighting shows the branches that connect the unique in Norfolk Island haplotype h143 with the most frequent haplotype worldwide, h131, and the haplotype h168 that is present in China and India. Only one mutation separates haplotype h143 from h131, while two mutations arose between this haplotype and the Chinese/Indian branch (h168).


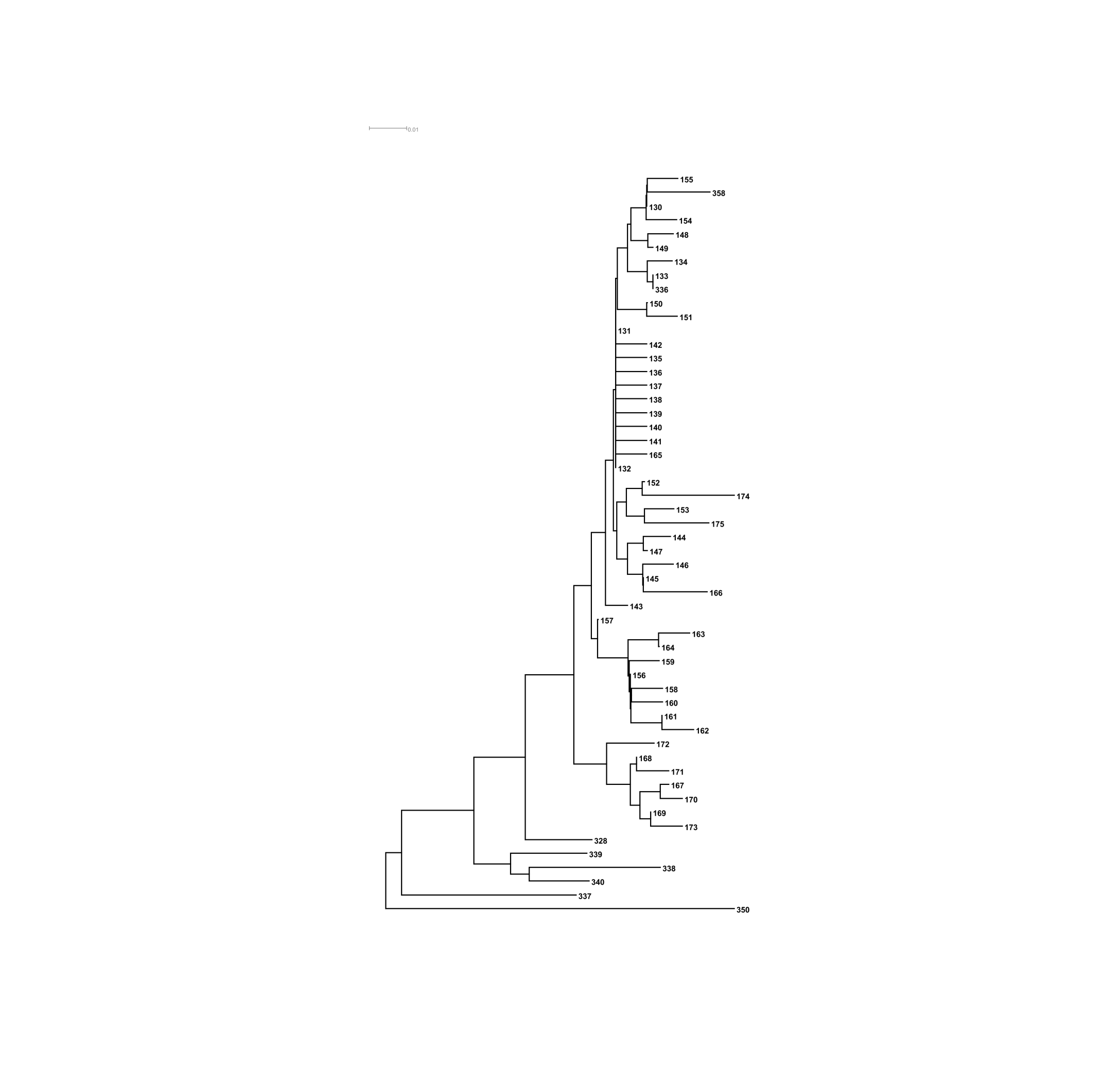


⬥

⬥

●

●

●

⬥

●

⬥

●

⬥

●

⬥

*0.01*

⬥


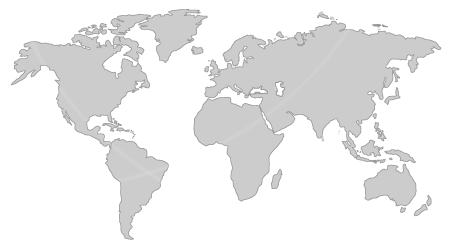

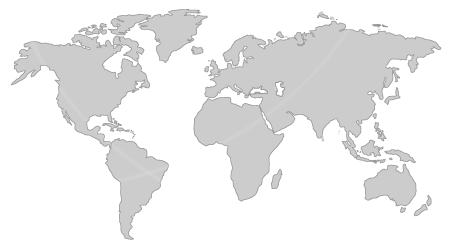

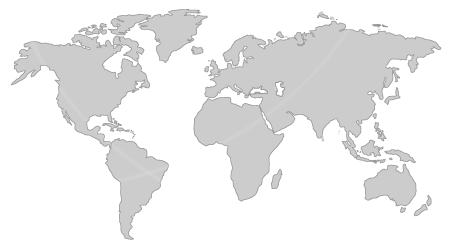


E1

E2

E3

E4

E5

**Figure S6B:** Neighbor-joining tree (K2P) for haplotypes within haplogroup E based on the 200 bp enriched dataset. Coloured boxes next to node names define the location where each haplotype is present. The world maps next to haplotypes 130, 131, and 146 indicate their widespread distribution, while haplotypes 143 and 145 are circled. Haplotype 143 is restricted to Norfolk Island feral chickens. Haplotype 145, which is present in China, Japan, India, Guam, and Australia, shows a narrower distribution than the rest of the Norfolk Island haplotypes.

**
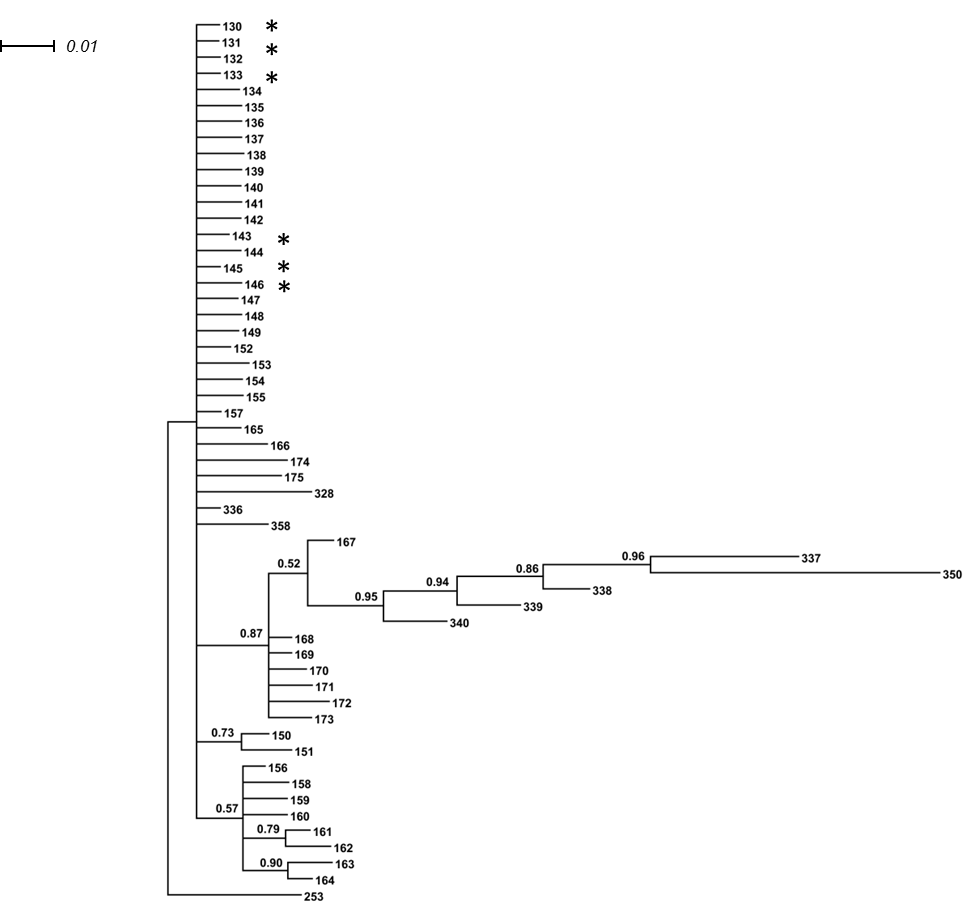
**

**Figure S6C:** Bayesian phylogenetic tree for haplogroup E based on the 200 bp enriched dataset. Numbers on clades show posterior probabilities. The sequence AF512158, belonging to haplotype 253, the main haplotype within haplogroup D, was used as an outgroup. The asterisk (*) indicates the haplotypes to which Norfolk Island chickens belong.
